# Prognostication of Three-Month Genicular Artery Embolization Outcomes Using Pre-Procedural MRIs

**DOI:** 10.1007/s00270-025-04159-8

**Published:** 2025-08-22

**Authors:** Wali Badar, Layth Alkhani, Faisal Al-Qawasmi, Ajay Varadhan, Ali Husnain, Mudassir Khan, Qian Yu, Magdalena Anitescu, Brendon Ross, Sara Wallace, Narayan Sundaram, Mikin Patel, Osman Ahmed

**Affiliations:** 1 Department of Radiology, University of Illinois at Chicago, 1740 West Taylor St, Chicago, IL USA; 2 Department of Engineering, Stanford University, Palo Alto, CA USA; 3 College of Medicine, University of Illinois at Peoria, Peoria, IL USA; 4 College of Medicine, University of South Florida, Tampa Bay, FL USA; 5 Department of Radiology, Northwestern Medicine, Chicago, IL USA; 6 College of Medicine, University of Illinois at Rockford, Rockford, IL USA; 7 Department of Radiology, University of Chicago, Chicago, IL USA; 8 Department of Anesthesia, University of Chicago, Chicago, IL USA; 9 Department of Orthopedic Surgery and Rehabilitation Medicine, University of Chicago, Chicago, IL USA

## Abstract

**Purpose:**

To assess the utility of pre-procedural knee MRI prior to genicular artery embolization (GAE) for the prognostication of early outcomes for symptomatic knee osteoarthritis (KOA).

**Materials and Methods:**

A single-center study including 39 patients received GAE from 9/2021–4/2025 with pre-procedural MRIs. MRIs were evaluated for structural abnormalities including menisci, ligaments, cartilage, marrow signal, and loose bodies. For 24 patients, synovitis was assessed using a semiquantitative method. Clinical outcomes were measured with the Western Ontario and McMaster Universities Osteoarthritis Index (WOMAC) scoring system at pre-GAE and three-month postintervention. Categorical response was assessed as a 50% reduction in the WOMAC Pain. Student t-tests were used to evaluate WOMAC pain reduction, and subset analysis was performed between MRI structural abnormalities and categorical response.

**Results:**

Fifty-two knees received GAE with pre-procedural MRIs (33 with contrast). There was a 34.6% clinical response rate (*N* = 8/52). Lateral meniscus and cartilage abnormality were predictive of poor categorical response at three months (*P* = 0.039–0.040). ≥ 4 structural abnormalities were associated with poor treatment response (*P* = 0.004). Pre-GAE synovitis was not predictive of categorical response at three months (*P* = 0.809). Kellgren–Lawrence ≥ 3 was predictive of poor response (*P* < 0.001). Lower adverse event rate was observed with temporary embolic compared to permanent embolic (*P* = 0.032).

**Conclusion:**

Pre-GAE knee MRI may offer short-term prognostic utility. Pre-procedural abnormalities in the lateral menisci and cartilage can predict poor response to GAE at three months. A greater degree of structural abnormality (≥ 4 structural abnormalities) was associated with poor response. Temporary embolic agent may be safer than permanent embolic agent.

**Graphic Abstract:**

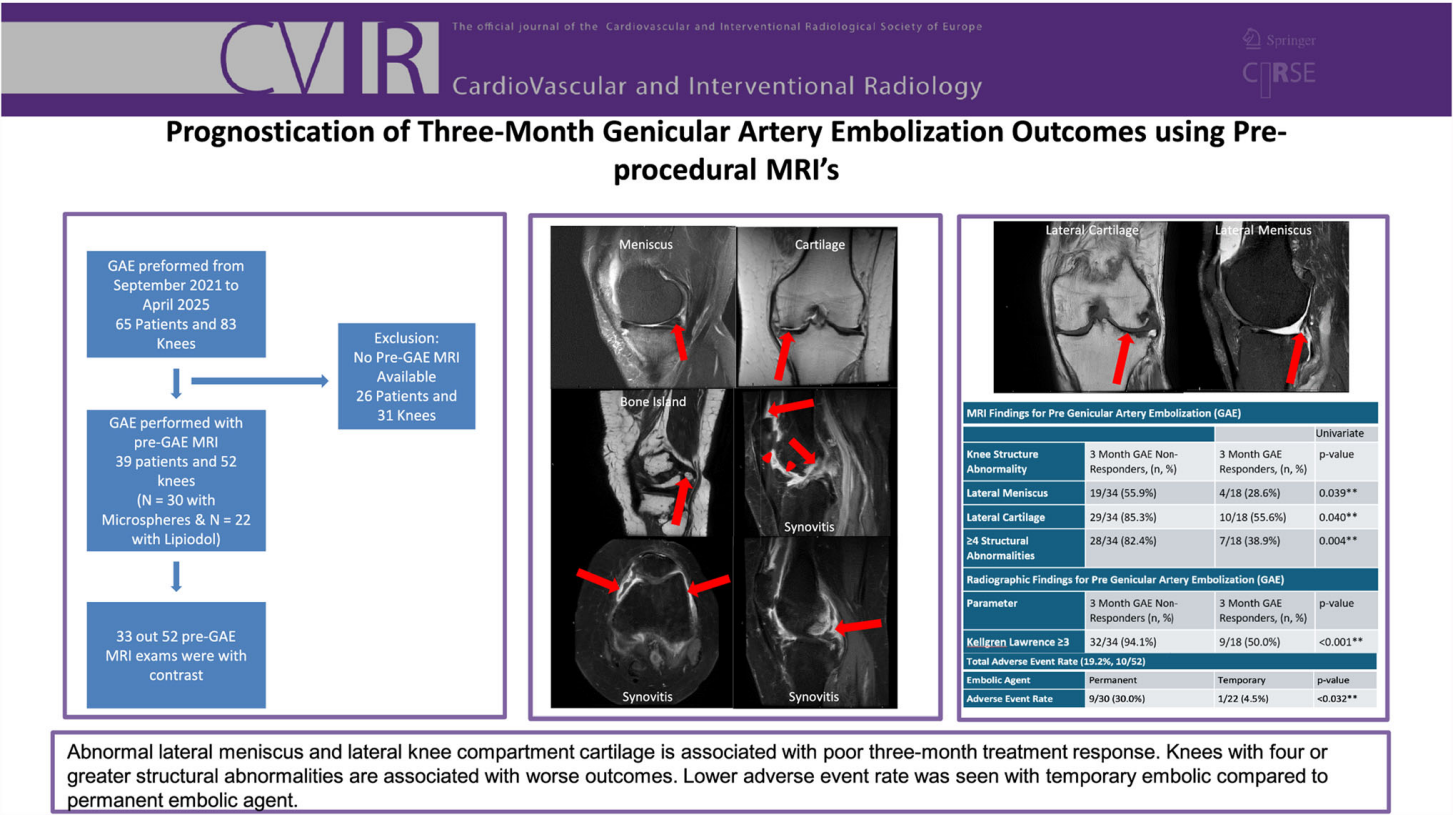

## Introduction

Knee osteoarthritis (KOA) is a leading cause of chronic disability among adults, affecting over 29.5 million people globally (1). Genicular artery embolization (GAE) has recently emerged as a treatment option for medically resistant KOA in poor surgical candidates or those looking to avoid or delay total knee arthroplasty. Since its first description by Okuno et al., multiple retrospective and prospective analyses have evaluated the safety and efficacy of this procedure (2, 3).

Steps to standardize pre-procedural imaging have largely remained underutilized, despite promising an increase in determining patient candidacy and grading. Based on plain radiographic findings (e.g., osteophytes, joint space narrowing, etc.), the commonly used Kellgren and Lawrence (KL) grading system is limited by its inability to detect nonosseous abnormalities that significantly contribute to OA-related knee pain. Specifically, radiographs are suboptimal for assessment of cartilage damage and correlate poorly with patients’ symptomatology (4).

Given the limitation of plain radiography, magnetic resonance (MR) imaging alternatively offers more comprehensive evaluation of nonosseous structures in and around the knee. Recently, few studies have shown potential as a predictive tool for treatment outcomes. Choi et al. evaluated pre-procedural noncontrast knee MRIs of 18 patients undergoing GAE for common imaging biomarkers of KOA and found that bone marrow lesions and meniscal injuries predicted poor treatment response at three-month post-GAE (5). Van Zadelhoff et al. investigated noncontrast pre-procedural MRIs of 44 patients undergoing GAE for imaging biomarkers associated with KOA in pre-procedural MRI and discovered that cartilage defects, bone marrow lesions, and osteophytes were predictive of treatment response at six-month post-GAE (6). Dablan et al. investigated contrast enhanced pre- and postprocedural MRI in 33 patients undergoing GAE and discovered that synovial enhancement decreases three months after GAE (7). While few studies have evaluated the utility of pre-procedural MRIs for GAE prognostication, further validation is necessary. Furthermore, this study aims to evaluate both contrast and noncontrast pre-procedural MRIs for short-term prognostication of GAE.

## Material and Methods

**Fig. 1:**
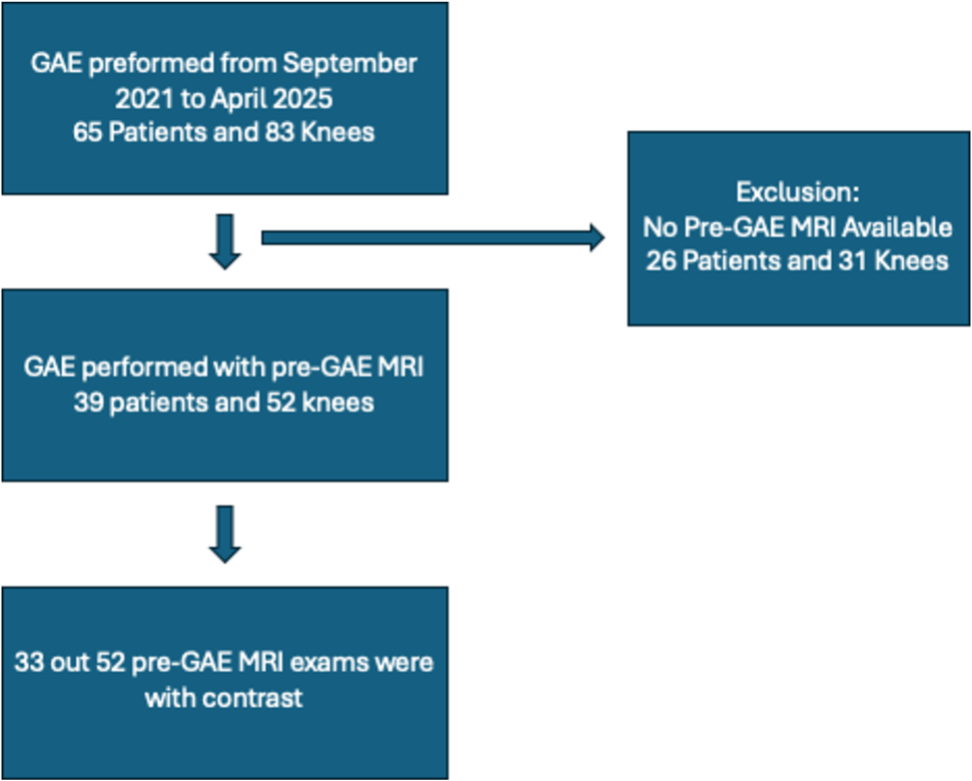
Summary of inclusion and exclusion criteria for study. GAE: genicular artery embolization

An institutional review board approved single-center retrospective review was conducted at a tertiary care academic hospital. Between September 2021 and April 2025, 65 consecutive patients (52 knees) underwent GAE (KL grade 1–4). Pre-procedural knee radiographs were reviewed by two board-certified musculoskeletal radiologists. Osteoarthritic pain refractory to medical management (> 6 months) was the primary indication for GAE. Of these 65 patients, 39 patients (52 knees) underwent pre-procedural imaging with MRI. Only patients with insurance approval for pre-GAE MRI received the MRI. Furthermore, only patients with insurance approval for contrast enhanced study received contrast enhanced studies (33 of 52 knees). Demographic information, procedural details, pre-procedural MRIs, and follow-up clinical data of these 39 patients were collected and analyzed. Inclusion and exclusion criteria are summarized in Fig. 1.

### GAE Procedure

An interventional radiologist with nine years of experience performed all GAE procedures. The procedural technique is described by the authors’ group elsewhere (BLINDED, 8). In brief, the embolization was performed using the pruning technique with permanent or temporary embolics. Permanent embolic included 200 μm microspheres (HydroPearl; Terumo) in 0.1 mL aliquots; the microspheres (5 mL) were suspended in 10 mL of iodinated contrast for a total embolic and contrast mixture of 15 mL. For temporary embolic, a combination of ethiodized oil and ioversol (Optiray 320, Guerbet) in a 3:1 ratio was used as a temporary embolic. The use of this combination has been previously described by Sapoval et al. (9). The volume of embolic material administered was recorded for each artery treated. In March 2024, there was an institutional transition to temporary embolic material and all subsequent GAEs were performed with the ethiodized oil and iversol combination. The switch was made due to the reported safety and efficacy of temporary embolics (9).

Future outpatient follow-ups were scheduled at 1 week, 1 month, and 3 months postprocedure. Total Western Ontario and McMaster Universities Osteoarthritis Index (WOMAC) and WOMAC pain scores were collected during the follow-up visits.

### MR Image Evaluation

The pre-procedural MRIs were reviewed independently by two board-certified musculoskeletal radiologists (5 and 8 years of experience, respectively) who were blinded to the clinical outcomes. The images were to be reviewed by a third musculoskeletal radiologist in case of interobserver disagreements; however, there were none. Standard imaging sequences included routine axial views (fat-saturated proton density [PD] turbo spin-echo [TSE]), sagittal views (T1-weighted TSE, T2-weighted TSE, and fat-saturated PD TSE), coronal views (fat-saturated T2-weighted TSE and PD TSE), and oblique–coronal views (T2-weighted TSE), all acquired with a slice thickness of 2–3 mm. For 33 out of 52 studies, postcontrast T1 fat-saturated axial and sagittal sequences were obtained after the administration of gadoteric acid, Dotarem**®** (Princeton, New Jersey, USA), based on the patient’s weight. Mean contrast volume administered is listed in Table 1. A majority (46 out of 52) of MRIs were performed on GE Discovery 750W 3.0 T machine (General Electric Company, Boston MA).

**Table 1:** Baseline Characteristics of Patients (*N* = 39)

Age, mean ± SD, *N* = 39		62 ± 14.7
Gender, *N* = 39 (%)	*Male*	9 (23)
	*Female*	30 (77)
Race, *N* = 29	*African American*	19 (49)
	*White*	19 (49)
	*Other*	1 (2)
Mean BMI ± SD (*N* = 39)		36 ± 10
Total number of knees treated, *N* = 52, n (%)	*Right*	28 (54)
	*Left*	24 (46)
KL grade, *N* = 52, n (%)	*Grade 2*	11 (21)
	*Grade 3*	23 (44)
	*Grade 4*	18 (35)
Duration (days) between index procedure and pre-intervention MR imaging, median (range)		19 (1–360)
MRI with Dotarem® intravenous contrast, *N* = 52, n (%)		33 (63)
Mean volume of Dotarem® administered (mL) ± SD		21.7 ± 5.3
Number of arteries embolized per procedure, mean ± SD		3 (0.9)
Volume (mL) of embolizing agent used per procedure, mean ± SD		1.9 (0.7)
Type of embolic used, *N* = 52, n (%)	Permanent (microspheres)	30 (58)
	Temporary (ethiodized oil + ioversol)	22 (42)
Embolized vessels, *N* = 169**,** n (%)	*Descending genicular artery*	35 (22)
	*Medial superior genicular artery*	15 (6)
	*Medial inferior genicular artery*	40 (25)
	*Lateral superior genicular artery*	33 (19)
	*Lateral inferior genicular artery*	44 (27)
	*Recurrent Anterior Tibial artery*	2 (2)

*BMI* Body Mass Index, *MRI* Magnetic Resonance Imaging, *KL* Kellgren–Lawrence

Knee pain was categorized into three compartments: lateral, medial, and patellofemoral. Radiologists assessed the images of each compartment for meniscal and cartilage defects, bone marrow edema, loose bodies, and cruciate ligament integrity. The findings were further categorized in a binary fashion as either “normal” or “abnormal.” Severity of each abnormality was not graded.

For postcontrast sequences (33 out 52 MRIs), synovial thickening was measured according to the semiquantitative method described by Guermazi et al. in the MOST study in the following nine locations: lateral and medial parapatellar space, suprapatellar space, infrapatellar space, intercondylar space, medial and lateral perimeniscal space, adjacent to the anterior and posterior cruciate ligaments, and adjacent to loose bodies or Baker’s cysts (10). Each location of enhancement was given a grade based on thickness (i.e., grade 0: < 2 mm, grade 1: 2–4 mm, and grade 3: > 4 mm). The total score was computed by adding the grade for each structure in a given knee.

### Outcomes

Technical success was defined as selective catheterization and successful embolization of at least one genicular artery that demonstrated abnormal synovial neo-angiogenesis and hyperemic blush. Primary clinical outcomes were measured numerically by percent reduction in WOMAC pain subscale score and categorically by: a 50% reduction in the WOMAC pain score at 3-month follow-up without any increase in baseline medication or intra-articular steroid use (11, 12).

Synovitis was graded using a semiquantitative scale described earlier on select patients with contrast enhanced MRIs (10).

The secondary outcome included the association between pre-procedural MRI’s findings and treatment results at 3-month follow-up. Adverse events were reported up to 1 month after GAE using Society of Interventional Radiology research reporting standards (13).

### Statistical Analysis

Patient demographics and procedural details were analyzed using descriptive statistics. Continuous variables were reported with mean and standard deviation. Time between pre-procedural MRI’s and GAE procedure was reported with median and range. Associations between categorical response or mean percent reduction in WOMAC pain scores and knee structural abnormalities assessed on MRI were assessed using Chi-square testing and ANOVA testing. Univariate analysis was performed using student t-tests. Correlation analysis was performed using Pearson correlation coefficient. Statistical testing was performed using MATLAB (MathWorks, Natick MA), and *p* < 0.05 was defined as statistical significance cutoff value.

## Results

### GAE and clinical outcomes

Treatment characteristics are summarized in Table 1. A total of 52 knees received GAE (R:L = 28:24) with 13 bilateral knee treatments. The Kellgren–Lawrence score distribution is as follows: Grade 2 (*n* = 11, 21%), Grade 3 (*n* = 23, 44%), Grade 4 (*n* = 18, 35%). Knees were treated with permanent microspheres (*n* = 30, 58%) and with ethiodized oil and ioversol emulsion (i.e., LipioJoint) (*n* = 22, 42%). A total of 169 vessels were embolized with an average of 3.0 ± 0.9 vessels treated per knee. The most commonly treated vessels were the descending genicular artery (*n* = 35, 22%), inferior medial genicular artery (*n* = 40, 25%), superior lateral genicular artery (*n* = 33, 19%), and inferior lateral genicular artery (*n* = 44, 27%).

The average baseline Total WOMAC score was 60.8 ± 17.3 with a 25.0% reduction at three-month post-GAE (*p* <  < 0.001). The average baseline WOMAC pain was 12.4 ± 3.3 with a 32.8% reduction at three-month post-GAE (*p* <  < 0.001). Categorical response, as defined by a 50% reduction in WOMAC pain with no increase in intra-articular steroid injection use, was 34.6% to GAE at 3 months. Clinical response outcomes are summarized in Table 2.

**Table 2:** Baseline and Posttreatment WOMAC (N = 52)

Characteristic	Mean ± SD	Range		*p*-value
Baseline Total WOMAC	60.8 ± 17.3	13–94		*p* < < 0.001
3-Month Total WOMAC	45.1 ± 18.4	8–81		
% Reduction from Baseline	25.0 ± 27.0			
Baseline WOMAC Pain	12.4 ± 3.3	4–20		*p* < < 0.001
3-Month WOMAC Pain	8.3 ± 4.5	1–20		
% Reduction from Baseline	32.8 ± 33.4			
Categorical Response after GAE (*N* = 52)				
Categorical Response	n		%	
50% WOMAC Pain Reduction at 3 Months	18		34.6	

*WOMAC* Western Ontario and McMaster Universities Osteoarthritis Index, *GAE* Genicular artery embolization

The categorical response rate at three months for temporary embolic was 45.4% (10 of 22) and for permanent embolic was 26.7% (8 of 30). No statistical difference was observed (*P* = 0.239). No difference in treatment response was observed based on the numbers of vessels treated (Responders: 3.3 ± 0.9 vs. Nonresponders: 3.2 ± 0.8, *P* = 0.616).

### Pre-procedural MRI Analysis

**Fig. 2:**
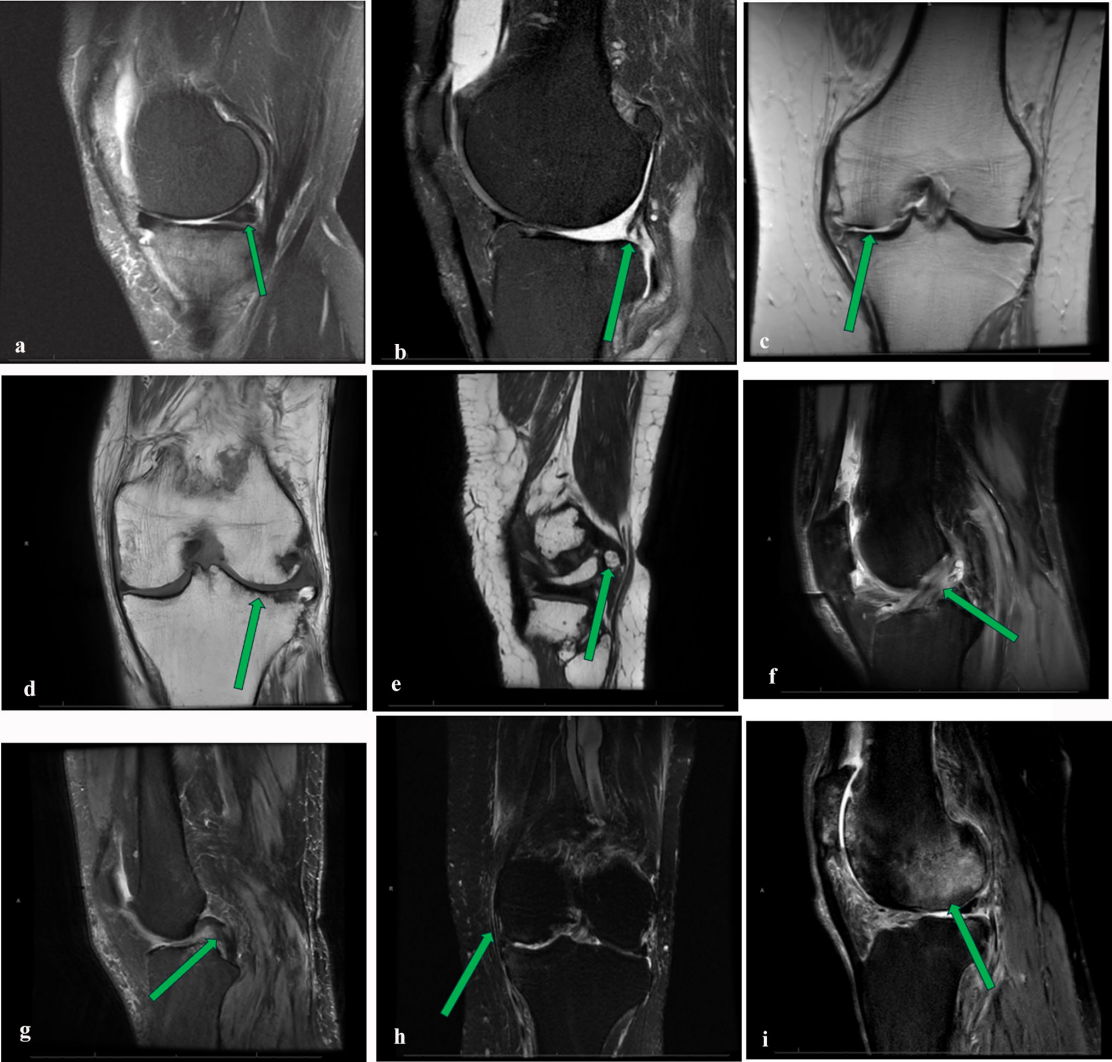
**a** Fluid-sensitive sagittal T2 fat-saturated image demonstrating complex tear of the posterior horn of the medial meniscus (arrow). **b** Fluid-sensitive sagittal proton density image demonstrating complex tearing and maceration of the posterior horn of the lateral meniscus (arrow). **c** T1 coronal image demonstrating full thickness cartilage defect in the medial compartment (arrow). **d** T1 coronal image demonstrating full thickness cartilage defect in the lateral compartment (arrow). **e** T1 coronal image demonstrating a posterior loose body (arrow). **f** Fluid-sensitive sagittal proton density image demonstrating mucoid degeneration of the anterior cruciate ligament (arrow). **g** Fluid-sensitive sagittal T2 fat saturation image demonstrating partial thickness tear of the posterior cruciate ligament (arrow). **h** Fluid-sensitive sagittal proton density image demonstrating mild sprain of the medial collateral ligament (arrow). **i** Fluid-sensitive sagittal proton density image demonstrating severe edema of the lateral femoral condyle (arrow)

**Fig. 3:**
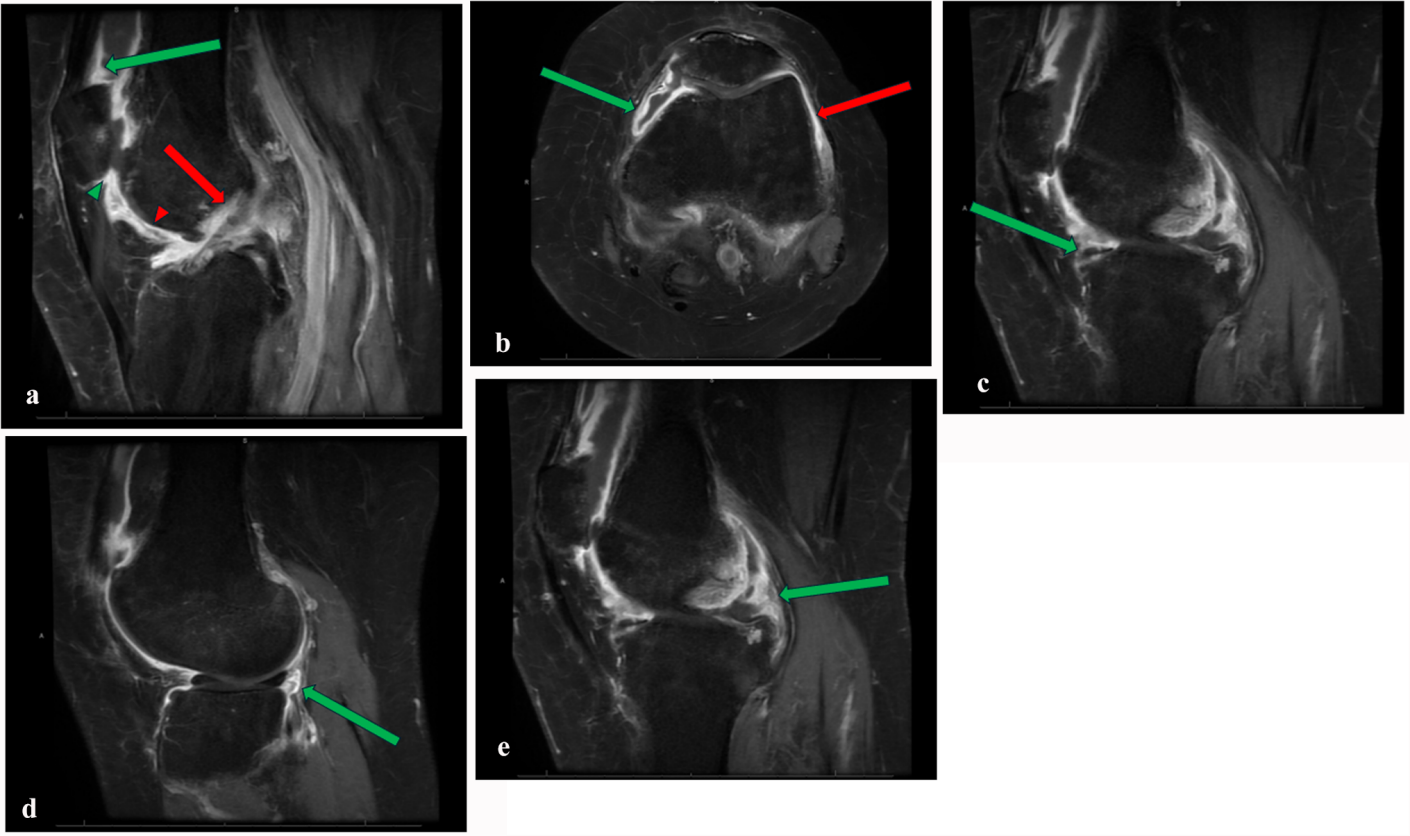
**a** T1 fat-saturated postcontrast sagittal image shows areas of synovitis that were measured in thickness. Suprapatellar is indicated by a blue arrow. Infrapatellar is indicated by a green arrow head. Adjacent to the anterior cruciate ligament is indicated by a red arrow. Intracondylar site is indicated by a red arrow head. **b** T1 fat-saturated postcontrast axial image shows areas of synovitis that were measured in thickness. Medial parapatellar is indicated by the green arrow and lateral parapatellar is indicated by the red arrow. **c** T1 fat-saturated postcontrast sagittal image shows areas of synovitis that were measured in thickness. Green arrow indicates medial parameniscal site of measurement. **d** T1 fat-saturated postcontrast sagittal image shows areas of synovitis that were measured in thickness. Green arrow indicates lateral parameniscal site of measurement. **e** T1 fat-saturated postcontrast sagittal image shows areas of synovitis that were measured in thickness. Green arrow indicates posterior cruciate ligament site of measurement

Of the ten imaging structures evaluated on pre-procedural MRIs: PCL, MCL, and LCL were excluded for statistical analysis because these structures were preserved across all knees. Samples of abnormal structures visualized are summarized in Fig. 2a–i. Incidence of knee structural abnormalities is summarized in Table 3. A majority of knees demonstrated medial meniscal (*n* = 34, 65.4%) compromise, medial (*n* = 45, 88.5%) and lateral (*n* = 39, 75.0%) cartilage loss, and bone marrow edema (*n* = 41, 78.8%). The mean synovitis score for postcontrast exams was 14.3 ± 3.7. Only three knees had no structural abnormalities. Areas where synovitis was graded are summarized in Fig. 3a–e.

**Table 3:** Pre-Procedural MRI Structural Lesions (N = 52)

Medial Meniscus (n,%)	34 (65.4%)
Lateral Meniscus (n,%)	23 (44.2%)
Medial Cartilage (n,%)	45 (88.5%)
Lateral Cartilage (n,%)	39 (75.0%)
Loose Bodies (n,%)	12 (23.1%)
ACL (n,%)	20 (38.5%)
PCL (n,%)	7 (13.5%)
MCL (n,%)	1 (1.9%)
LCL (n,%)	0 (0%)
Bone Marrow Edema (n,%)	41 (78.8%)
Mean Synovitis ± SD (*N* = 33)	14.3 ± 3.7

*ACL* Anterior Cruciate Ligament, *PCL* Posterior Cruciate Ligament, *MCL* Medial Collateral Ligament, *LCL* Lateral Collateral Ligament

### Pre-procedural MRI and Radiographs Correlated with GAE Clinical Outcomes

An association was observed between abnormal lateral menisci and three-month categorical response (*P* = 0.039) and abnormal lateral cartilage (*P* = 0.040) (Table 4). No additional associations were observed between individual abnormal knee structures and three-month response. Knees with four or more structural abnormalities were associated with poor three-month categorical response (*P* = 0.004).

**Table 4:** MRI Findings

			Univariate
Knee Structure Abnormality	3-Month GAE Nonresponders, *N* = 34, (n, %)	3-Month GAE Responders, *N* = 18, (n, %)	*p*-value
MM	25 (73.5%)	9 (50.0%)	0.128
LM	19 (55.9%)	4 (28.6%)	0.039**
MC	31 (91.1%)	14 (71.4%)	0.218
LC	29 (85.3%)	10 (55.6%)	0.040**
LB	7 (20.6%)	5 (27.8%)	0.731
ACL	16 (47.1%)	4 (28.6%)	0.134
BE	29 (85.3%)	12 (66.7%)	0.159
≥ 4 Structural Abnormalities	28 (82.4%)	7 (38.9%)	0.004**

Significance level is *p* < 0.05

*GAE* Genicular artery embolization, *MM* Medial Meniscus, *LM* lateral meniscus, *MC* medial compartment cartilage, *LC* lateral compartment cartilage, *LB* loose bodies, *ACL* Anterior Cruciate Ligament, *BE* Bone Marrow Edema, *KL* Kellgren–Lawrence

On univariate analysis, there was no association with three-month categorical response and synovitis score (Responders: 14.0 ± 2.8 vs. Nonresponders: 14.4 ± 4.1, *P* = 0.809). No correlation was observed between synovitis score and three-month WOMAC pain reduction (ρ = −0.135, *P* = 0.457).

An association was observed between radiographic severity of KOA (KL ≥ 3) and three-month categorical response (*P* < 0.001) (Table 5).

**Table 5:** Radiographic Findings

Parameter	3-Month GAE Nonresponders, *N* = 34, (n, %)	3-Month GAE Responders, *N* = 18, (n, %)	*p*-value
KL ≥ 3	32 (94.1%)	9 (50.0%)	< 0.001**

Significance level is *p* < 0.05

The most common adverse event was skin changes without ulceration (*n* = 9, 17.3%), knee swelling (*n* = 3, 5.8%), and hematoma (*n* = 1, 1.9%). All adverse events were self-resolving and considered mild. In total, 10 out 52 knees (19.2%) had post-GAE adverse events. No association was identified between adverse events and knee structures evaluated on MRI (*p* = 0.136–0.999) or synovitis (*P* = 0.319). Lower adverse event rate was observed with temporary embolic compared to permanent embolic (4.5% vs. 30%, *P* = 0.032).

## Discussion

Although previous studies highlight its potential utility, pre-procedural cross-sectional imaging with MRI is not well described for treatment planning and prognostication in GAE. Gill et al. evaluated outcomes of GAE on 33 patients with mild versus moderate to severe knee OA as evaluated by the KL grading system using the Knee Injury and Osteoarthritis Outcomes Score (KOOS). The authors found that patients with KL grade 2 responded more favorably to GAE to KL grade 3 and 4 at six-month post-GAE (14). Choi et al. investigated the benefits of pre-procedural MRIs for predicting GAE treatment response at one and three months using the visual analog scale (VAS) in 18 patients and 28 knees (5). They found that the KL scale along with bone marrow edema and meniscal injury predicted poor treatment response. Dablan et al. evaluated knee synovitis on pre- and postprocedural contrast enhanced knee MRI in 33 patients and 35 knees. The authors discovered that the degree of pre-procedural synovial enhancement correlated with pain relief at three-month post-GAE, based on changes in VAS score but not the WOMAC pain score (7). The current study agreed with the findings of Gill et al. and Dablan et al. with regard to radiographic severity of KOA (KL ≥ 3) predicting poor treatment response.

The current study serves as further evidence supporting the utility of pre-procedural MRIs for prognostication of GAE. Similar to the Choi et al. study, this study identified meniscal compromise as a predictor of categorical response after GAE. Specifically, abnormal lateral menisci predicted poor response at three months. Interestingly, abnormal medial meniscus was not a predictor of treatment response although knee OA is often more severe in the medial compartment (15). However, Roubille et al. (16) evaluated meniscal lesions in knee OA and discovered that lateral meniscal lesions are a strong predictor or neuropathic pain in symptomatic knee OA although a mechanism is not clear. Additionally, the current study uncovered that lateral compartment cartilage loss was associated with poor treatment response. This finding corroborated the findings of van Zadelhoff et al. (6) which identified cartilage defects as a predictor of less reduction in WOMAC pain scores after GAE. A majority of knees with lateral meniscal injury also had lateral cartilage loss (18 out of 23). This may suggest that there is subchondral bone on bone contact resulting in pain that is not responsive to GAE. Lastly, the burden of knee structural compromise (i.e., ≥ 4 structural abnormalities) was associated with poor treatment response. This may be explained by the fact that the factors investigated in this study are linked to KOA progression and thus a high burden of structural abnormality may indicate advanced KOA that is treatment resistant to GAE (17–21). Furthermore, this study evaluated the same synovitis scoring method as Dablan et al. (7) and found that similarly pre-procedural synovial enhancement did not have correlation with WOMAC pain reduction. In contrast to Dablan et al., change in synovitis score after GAE was not assessed and the VAS score was not used for analysis in the current study.

Although the intent of this study was to evaluate the prognostic capability of pre-procedural MRIs, the current study investigated the efficacy of lipiodol as a temporary embolic for GAE. Few studies have evaluated lipiodol, but the current study agrees principally with previous studies in that lipiodol was effective for achieving clinical response (9, 22). A low adverse event profile was observed with lipiodol similar to previously published studies. Additionally, the adverse event rate for lipiodol was lower than microspheres.

This study had several limitations, including those typical of retrospective studies with small sample sizes, which limited the generalizability of the findings. The limited sample size prevented the ability to perform subset analysis. Not all patients received pre-GAE MRIs due to insurance approval introducing bias. Additionally, the severity of each abnormal structure was not graded and a standardized scale was not used to evaluate the knee. Not all patients received IV contrast for their MRIs, weakening the generalizability of the prognostic utility of synovitis as well as introducing selection bias. Future studies with standardized imaging protocols are necessary to reduce selection bias as not every patient received pre-GAE MRIs. This can also address variability that may exist with using multiple MRI scanners (i.e., manufacturer variability and field strength). Most subjects did not receive postprocedural MRIs, and therefore, correlation of symptoms with changes in imaging biomarkers was not possible. Heterogeneity in embolic selection, i.e., permanent microspheres and lipiodol-contrast emulsion, may also serve as a potential confounder. Future studies with larger sample sizes and uniform treatment protocols can elucidate relationships between measured parameters and treatment response. Similar to previous studies, the current study only included short-term follow-up for up to 3-month post-GAE. Future studies with longer follow-up (12 and 24 months) will shine light on long-term prognostic capabilities of pre-procedural imaging. The study focused on WOMAC pain for statistical analysis, and therefore, the findings are not generalizable to other pain assessment scales such as VAS or KOOS. Lastly, this study did not evaluate how concurrent analgesia usage changed (i.e., NSAIDs or opioids).

Symptomatic knee pain is a multifactorial process that includes osteogenic and nonosteogenic etiologies. Osteogenic etiologies can be assessed on radiographs; however, nonosteogenic factors such as meniscal injury or synovitis can be radiographically occult. MRI can help detect nonosteogenic factors that contribute symptomatic knee osteoarthritis and thus can serve as an important tool for GAE treatment selection. The current study identified that meniscal and cartilage injury along with burden of injury may be a predictor of categorical response after GAE in patients with knee OA which may aid in optimizing patient selection for this treatment. Lastly, temporary embolic agents may be safer than permanent embolic agents; however, future large-scale head-to-head studies are needed to understand the comparative performance.
